# Substance-Related Health Problems during Rave Parties in the Netherlands (1997–2008)

**DOI:** 10.1371/journal.pone.0029620

**Published:** 2011-12-28

**Authors:** Jan Krul, Matthijs Blankers, Armand R. J. Girbes

**Affiliations:** 1 Educare Groningen, Groningen, The Netherlands; 2 Department of Psychiatry, Arkin and Amsterdam Institute for Addiction Research, Academic Medical Center, University of Amsterdam, Amsterdam, The Netherlands; 3 Department of Intensive Care, University Hospital VU Medical Centre, Amsterdam, The Netherlands; Finnish Institute of Occupational Health, Finland

## Abstract

The objective of this study was to describe a 12-year (1997–2008) observation of substance-related incidents occurring at rave parties in the Netherlands, including length of visits to first-aid stations, substances used, and severity of the incidents. During rave parties, specifically trained medical and paramedical personnel staffed first aid stations. Visitors were diagnosed and treated, and their data were recorded using standardized methods. During the 12-year period with 249 rave parties involving about 3,800,000 visitors, 27,897 people visited a first aid station, of whom 10,100 reported having a substance-related problem. The mean age of these people was 22.3+/−5.4 years; 52.4% of them were male. Most (66.7%) substance-related problems were associated with ecstasy or alcohol use or both. Among 10,100 substance-related cases, 515 required professional medical care, and 16 of these cases were life threatening. People with a substance-related problem stayed 20 min at the first aid station, which was significantly longer than the 5 min that those without a substance-related health problem stayed. These unique data from the Netherlands identify a variety of acute health problems related to the use of alcohol, amphetamines, cannabis, cocaine, ecstasy, and GHB. Although most problems were minor, people using GHB more often required professional medical care those using the other substances. We recommended adherence to harm and risk reduction policy, and the use of first aid stations with specially trained staff for both minor and serious incidents.

## Introduction

In the early 1990s, a new music culture called *dance* spread through numerous countries in the western world. Rave parties or house parties with DJ-directed, fast-paced electronic music and light shows were organized [1],[2]. In The Netherlands, rave parties attract from 500 to 60,000 visitors. Approximately 650,000 youngsters (15–35 year olds) [3] attend these events yearly. Several studies have indicated that the use of recreational substances during raves is common, and the majority of visitors use one or more substances [1],[4]–[13].

In the Netherlands, rave parties are allowed only if strict regulations are met. One of these is that a first aid station is required to take care of rave party attendees with various health-related problems. Here we present an overview of substance-related visits to first aid stations at rave parties in the Netherlands between 1997 and 2008.

### Substance Use in the Netherlands

In 1997, 2001, 2005, and 2009, surveys of substance use in the general population in the Netherlands were conducted. Each of the substances covered in the present study were included in these surveys. During this period, lifetime prevalence of cannabis use generally increased (from 19.1% in 1997 to 25.7% in 2009), as did ecstasy use (from 2.3% to 6.2%) and cocaine use (from 2.6% in 1997, 2.1% in 2001, 3.4% in 2005, to 5.2% in 2009). For the first time in 2009, lifetime prevalence of γ-Hydroxybutyric acid (GHB) use was determined to be 1.3%, and the lifetime prevalence of alcohol use was 84% [14]. In this study, we describe substance-related health problems that occurred during rave parties.

## Methods

This was a prospective observational study of rave-party attendees who presented themselves for help at first aid stations at rave parties during the period 1997–2008. All persons seeking first aid were registered, but only those with substance-related problems were included in this study. Health-related incidents are described, together with length of medical care, severity of the incidents, predictability of symptoms, and short-term risks. Specifically, the following information was collected with regard to rave party attendees who sought help at first aid stations: (1) Length of stay, (2) substances used alone or in combination, and (3) nature of the substance-related problems.

In an unpublished 1996 prospective pilot study of rave-party attendees, those seeking first aid were divided into two groups. The first was a self-care group. These people visited a first-aid station with only minor health-related problems and were not included in the study. The second group visited the first aid station seeking help or advice. For this group, a standard questionnaire was developed to ask about their health-related problems [7]. Each person's age and sex and time of arrival at and departure from the first aid station were recorded. Additional questions asked about their substance use and referrals that had been made to a general practitioner, dentist, or hospital. Finally, based on the Emergency Severity Index [15],[16], each health-related incident was categorized as medical, traumatic, psychological, or miscellaneous, and each incident was designated as minor, moderate (defined as requiring professional medical care within six hours), or severe (defined as life-threatening and needing immediate professional medical care).

In the present observational study, data were collected prospectively and anonymously. According to Dutch regulations, neither medical nor ethical approval was needed to conduct the study. The study was not supported financially in any way. The data were obtained from files maintained by Educare, a nonprofit organization that provides first-aid assistance at large-scale events. The Educare Board of Directors consented to our using the data for scientific purposes.

### Procedure

Upon entering the first aid station, the person was seen by a clerical officer, who determined whether medical assistance was necessary or self-care was sufficient. If aid was required, the person was referred to a member of the medical staff. This staff included qualified nurses, paramedics, and physicians, all of whom had received training in rave-related health risks, including the effects of psychoactive substances. They had also been trained to use the standardized questionnaire. An experienced co-worker was appointed to assist the staff in filling out the questionnaires, and this person coached all of the staff members in using the questionnaires. After the rave-party attendee had been discharged from the first aid station, the co-worker checked all of the data to verify their integrity.

The number of visitors to each rave party (i.e., the number of tickets sold) was obtained from the organizers of the event. Serious health-related incidents were defined as those rated as *moderate* or *severe* on the Emergency Severity Index [15]. Risk of a serious incident from each substance was defined as the number of serious incidents that occurred divided by the number of attendees who used that substance. Relative risk (RR) of a serious incident from each substance was defined as risk of a serious incident from that substance divided by risk of a serious incident for visitors seeking first aid who did not report using the substance. For each substance, the likelihood of visiting a first-aid station was calculated by dividing the number of users of that substance who sought first aid by the total number of visitor who sought first aid.

### Statistics

Descriptive statistics were used to understand the demographic characteristics of the sample and the nature of their substance-related visits to first-aid stations. To evaluate the statistical significance of the results, Person's *r* and Mann-Whitney *U* tests were used for the parametric and nonparametric data, respectively. A *p* value of <0.05 was the cut-off for significance. To explore relationships between health-related incidents and substance use, an logistic regression analysis was performed. A stepwise forward regression model was used, with *P*(in) = 0.05 and *P*(out) = 0.10; a maximum of 20 iterations was specified and a cut-off value of 0.5; the predictors of health-related incidents were added using a stepwise procedure. The models were evaluated for acceptable fit and proportions of variance explained. From each of the specified models, odds ratios >2 are presented. All analyses were performed using SPSS version 17.0.

## Results

From 1997 to 2008, 3,793,500 visitors attended 249 rave parties. Most (70%) of the raves occurred at night. Many (*N* = 27,897; 0.7% of all visitors) of the people visiting a first-aid station presented with complaints that needed some form of medical attention. The mean age of all people visiting first-aid stations was 22.3 years (*SD* = 5.4), but the age of the visitors increased significantly from 18.7 years in 1997 to 24.0 years in 2008 (*r* = .245, *p*<.01). Visitors seeking help at first-aid stations were approximately equally divided between males (52.4%) and females (47.0%).

Across the 12 years, a total of 10,100 people (36.2% of those seeking first aid), representing 0.3% of all rave party visitors, experienced a substance-related incident. The incidents were medical (80%), traumatic (9%), psychological (4%), or miscellaneous (7%). The median overall length of stay at a first aid station was 10 minutes, but it was 20 minutes for substance-using visitors and 5 minutes for nonsubstance-using visitors—a difference that is statistically significant (*p*<.001) (Table 1).

**Table 1:** Characteristics of visitors at first aid stations.

Year	Nr of FAA	Substance use%	Mean Age(SD)	Sex M %***	Sex F %***	General Stay at FAS* Median (Range)	Stay at FAS Substance-related* Median (Range)	Stay at FASNot substance-related* Median (Range)
**1997**	2044	57.6	18.7 (2.8)	62.0	37.4	12 (294)	15 (294)	7 (137)**
**1998**	1566	41.4	20.0 (4.2)	52.4	46.4	10 (389)	18 (389)	6 (184)**
**1999**	1683	39.9	21.0 (4.5)	56.3	43.2	10 (361)	20 (197)	8 (361)**
**2000**	1843	36.4	22.2 (5.1)	53.0	46.7	7 (359)	15 (264)	5 (359)**
**2001**	3629	37.8	21.7 (4.8)	50.3	49.1	5 (272)	13 (272)	4 (266)**
**2002**	2971	37.3	22.0 (5.1)	52.6	47.3	10 (294)	15 (294)	5 (248)**
**2003**	3337	34.0	22.5 (5.2)	51.8	47.9	10 (269)	20 (269)	6 (163)**
**2004**	3818	30.5	23.5 (5.8)	53.4	46.3	10 (323)	20 (241)	5 (323)**
**2005**	2690	34.0	23.3 (5.7)	50.1	48.8	10 (312)	23 (312)	6 (216)**
**2006**	1249	28.1	23.4 (5.5)	46.1	52.8	10 (364)	25 (293)	8 (364**
**2007**	1600	27.8	24.0 (6.5)	48.1	51.6	10 (241)	28 (212)	8 (241)**
**2008**	1467	30.1	24.0 (6.3)	52.3	47.4	10 (554)	30 (274)	7 (554)**
**Total**	27897							
**Mean**		36.2	22.3 (5.4)	52.4	47.0			
**Median**						10 (554)	20 (389)	5 (554)**

*in minutes.

**p<0.001, compared to substance-related visits to first aid stations.

***missing data.

Most (*n* = 6912, 64.4%) of the substance-using visitors reported using only one substance, which was usually ecstasy (*n* = 3308, 32.8%) or alcohol (*n* = 2296, 22.7%), but a substantial proportion (*n* = 2554, 25.3%) reported using two substances simultaneously. The most commonly reported combined use was ecstasy with alcohol (*n* = 1129, 11.2%) (Table 2).

**Table 2:** Number of individual and multiple substance-using first aid visitors.

N = 10,100	Alc	Amp	Can	Coc	GHB*	Ecs	Total
**Single users** **	**2296** **(22.7%)**	**331** **(3.3%)**	**190** **(1.8%)**	**44 (0.4%)**	**252** **(2.5%)**	**3308** **(32.8%)**	**6912 (68.4%)**
**Double users**							2554(25.3%)
**Alc**	-	70 (0.7%)	384 (3.8%)	47 (0.5%)	123 (1.2%)	1129 (11.2%)	
**Amp**	70 (0.7%)	-	5 (0.0%)	13 (0.1%)	18 (0.2%)	428 (4.2%)	
**Can**	384 (3.8%)	5 (0.0%)	-	8 (0.1%)	61 (0.6%)	6 (0.1%)	
**Coc**	47 (0.5%)	13 (0.1%)	8 (0.1%)	-	6 (0.1%)	66 (0.7%)	
**GHB**	123 (1.2%)	18 (0.2%)	61 (0.6%)	6 (0.1%)	-	190 (1.9%)	
**Ecs**	1129 (11.2%)	428 (4.2%)	6 (0.1%)	66 (0.7%)	190 (1.9%)	-	

Alc = alcohol. Amp = amphetamines. Can = cannabis. Coc = cocaine. Ecs = ecstasy.

*GHB was monitored after the year 2000.

**Magic mushrooms (*n* = 35). Unidentified products from smartshops such as energizers (*n* = 229), medication (*n* = 127), and other unidentified substances (*n* = 223) were excluded.

The most common substance-related health problem was a general feeling of being unwell/fainting, which was associated with the use of all substances. Additional minor health-related problems were associated with the different substances. Using amphetamines was associated with having cramps (OR = 6.9); cocaine, with having a high body temperature (>37.5°C) (OR = 29.5) or palpitations (OR = 6.5); and GHB, with altered consciousness (OR = 32.7) (Table 3). Although ecstasy is a stimulant, the combined use of ecstasy and GHB was associated with having a subnormal body temperature (OR = 5.6). The combined use of ecstasy and amphetamines were associated with having psychotic delusions (OR = 9.7), high body temperature (OR = 5.5), cramps (OR = 4.2), palpitations (OR = 3.4), or a stomachache (OR = 2.8). The highest odds ratios were found for the association between altered consciousness and GHB/ecstasy use (OR = 26.2) and GHB/alcohol use (OR = 25.3) (Table 4).

**Table 3:** Most common individual substance-related incidents and odds ratio (OR) among first aid station visitors (%).

	Alc	*OR*	Amp	*OR*	Can	*OR*	Coc	*OR*	GHB	*OR*	Ecs	*OR*
	N = 2296	*(CI)*	N = 331	*(CI)*	N = 190	*(CI)*	N = 44	*(CI)*	N = 252	*(CI)*	N = 3308	*(CI)*
**Altered consciousness**	4.7		3.3		5.3		0.0		56.0	*32.7 (20.7–51.6)*	3.8	*0.75 (0.60–0.95)*
**Body temperature <36.5°C**	2.6	*2.3 (1.2–4.2)*	0.9		0.5		0.0		22.2	*3.4 (1.5–7.3)*	1.2	*0.2 (0.1–0.5)*
**Body temperature >37.5°C**	0.3		0.3		1.6		4.5	*29.5 (6.7–130.9)*	0.8		0.8	
**Cramps**	1.2	*0.3 (0.2–0.6)*	19.0	*6.9 (5.0–9.7)*	0.5		4.5		2.0		5.3	*1.7 (1.4–2.1)*
**Delusions**	0.3	*0.3 (0.1–0.8)*	6.0	*5.3 (3.0–9.2)*	0.0		2.3		0.8		2.0	*2 (1.4–2.9)*
**Dizziness**	15.6	*1.3 (1.0–1.5)*	12.3		27.9	*4.1 (2.7–6.3)*	11.4		6.0		18.0	*1.7 (1.5–2.0)*
**Generally unwell/fainting**	34.8		40.4	*2.1 (1.6–2.7)*	37.4		34.1		34.5		52.8	*5.1 (4.6–5.6)*
**Nausea**	22.8	*1.5 (1.3–1.8)*	21.4	*1.8 (1.3 2.5)*	31.6	*1.8 (1.1–2.9)*	9.1		14.3		25.4	*2.3 (2.0–2.6)*
**Palpitations**	1.2	*0.4 (0.2–0.8)*	7.5	*3.6 (2.0–6.3)*	3.7		13.6	*6.5 (1.5–28.4)*	0.0		2.5	*1.5 (1.1–2.1)*
**Stomach ache**	3.5		6.3	*2.6 (1.6–4.4)*	1.6		4.5		0.8		3.1	*1.6 (1.2–2.1)*
**Vomiting**	16.8	*2.1 (1.7–2.6)*	6.9		15.8	*1.9 (1.1–3.5)*	6.8		17.5		10.9	

Alc = alcohol. Amp = amphetamines. Can = cannabis. Coc = cocaine. Ecs = ecstasy.

More than one symptom can occur in combination with each substance.

Reference category for the logistic regression analysis is not reporting use of each of the substances.

95% CI = 95% confidence interval (lower-upper).

ORs are available only for variables included in the forward stepwise model.

**Table 4:** Most common multiple-substance-use problems and odds ratios (ORs) among first-aid station visitors (%).

	Alc+Ecs	*OR (CI)*	Amp+Ecs	*OR (CI)*	Alc+Can	*OR (CI)*	GHB+Ecs	*OR (CI)*	Alc+GHB	*OR (CI)*
	N = 1129		N = 428		N = 384		N = 190		N = 123	
**Altered consciousness**	8.1	*1.8 (1.3–2.5)*	5.1		7.8	*2.6 (1.6–4.2)*	52.1	*26.2 (16.3–42.2)*	56.9	*25.3 (12.7–50.2)*
**Anxiety**	7.1		10.3		2.6		1.6		0.8	
**Body temperature <36.5°C**	3.1		0.2		3.4		21.1	*5.6 (2.7–11.7)*	20.3	
**Body temperature >37.5°C**	1.2		2.1	*5.5 (2.2–13.6)*	0.3		2.1		3.3	
**Cramps**	3.0		14.3	*4.2 (3.1–5.9)*	1.0		3.7		0.0	
**Delusions**	1.7		8.9	*9.7 (6.3–14.9)*	0.5		0.5		0.0	
**Disorientation**	5.4	*4.1 (2.7–6.1)*	0.9		2.6		7.9		8.9	*4.3 (1.9–9.3)*
**Dizziness**	16.1		13.3		25.7	*2.3 (1.5–3.3)*	3.7	*0.2 (0.0–0.8)*	6.5	
**Generally unwell/fainting**	57.2	*3.5 (2.9–4.3)*	65.2	*5.9 (4.7–7.4)*	48.8	*1.5 (1.1–2.1)*	43.7		35.0	
**Nausea**	24.1	*1.7 (1.3–2.1)*	22.9	*1.6 (1.2–2.0)*	41.3	*2.1 (1.4–3.1)*	11.1		13.0	
**Palpitations**	3.5	*2.2 (1.4–3.7)*	6.5	*3.4 (2.1–5.6)*	1.3		0.5		1.6	
**Stomachache**	2.2		5.8	*2.8 (1.8–4.5)*	2.1		0.5		2.4	
**Vomiting**	13.4		9.1		30.9	*3.2 (2.1–4.8)*	13.2		21.1	

Alc = alcohol. Amp = amphetamines. Can = cannabis. Coc = cocaine. Ecs = ecstasy.

More than one symptom can occur in combination with the two combined substances.

Reference category for the logistic regression analysis is not reporting the use of each combination of the substances.

95% CI = 95% confidence interval (lower-upper).

ORs are available only for variables included in the forward stepwise model.

The number of rave-party visitors who sought first aid fluctuated during the 12 years of the study. Between 1997 and 2000, 7,136 people (1.0% of the visitors) needed first aid. Between 2001 and 2004, the number rose to 13,755 (0.8% of the visitors). From 2005 to 2008, 7,006 visitors sought first aid. Across the 12 years, a total of 515 cases were considered serious (i.e., professional medical care was required; Category 1 and 2 of the Severity Index [15]), and 262 of these were admitted to a hospital emergency room. First aid stations, however, were mostly often visited by people with no substance-related health complaint. Among those who did use substances, the risk of a serious incident was highest among ecstasy users in the period 1997–2000 (0.21), but this risk decreased to 0.06 in 2005–2008. For alcohol users, the risk was 0.06 during 1997–2000, but it increased to 0.09 in 2001–2004 and 2005–2008 (Table 5). In 2001–2004, the relative risk of having a cocaine-related incident was 21.0. The largest number of serious incidents occurred with GHB use (*N* = 55), with relative risk of 31.9 between 2001–2004 and 48.9 between 2005–2008 (Table 5). Serious incidents also occurred in these periods with GHB/alcohol use (*N* = 32; relative risk = 41.4 and 50.5) and GHB/ecstasy use (*N* = 47, relative risk = 44.1 and 44.8) (Table 6). The combined use of alcohol and cannabis was not associated with having a problem. On the whole, the relative risk of having a serious incident was higher among substance-using visitors needing first aid compared to those who were not using a substance (Tables 5–6). Finally, all severe incidents (life-threatening, Category 1 of the Severity Index [15], *N* = 16) were substance-related; they included four cases of excited delirium, and three each of circulatory insufficiency, respiratory insufficiency, hyperthermia, and severe trauma.

**Table 5:** Number and risk of first-aid visits and serious incidents associated with using different substances individually.

Substance	Period	Nr of FAVs	Risk FAVs	Nr of SI	RR SI (CI)	Risk SI
**alcohol**	1997–2000	401	0.06	6	1.9 (0.8–4.4)	0.02
	2001–2004	1281	0.09	18	2.3 (1.3–3.8)	0.01
	2005–2008	616	0.09	8	2.4 (1.1–5.2)	0.01
**cannabis**	1997–2000	46	0.01	1	2.7 (0.4–19.4)	0.02
	2001–2004	110	0.01	0	0 (0.0–0.0)	0
	2005–2008	34	0.01	0	0 (0.0–0.0)	0
**cocaine**	1997–2000	12	0	0	0 (0.0–0.0)	0
	2001–2004	23	0	3	21 (7.1–62.2)	0.13
	2005–2008	9	0	0	0 (0.0–0.0)	0
**ecstasy**	1997–2000	1487	0.21	15	1.3 (0.7–2.3)	0.01
	2001–2004	1405	0.1	10	1.1 (0.6–2.2)	0.01
	2005–2008	418	0.06	6	2.6 (1.1–6.3)	0.01
**GHB**	1997–2000	12	0	2	20.8 (5.6–77.1)	0.17
	2001–2004	136	0.01	27	31.9 (20.8–48.9)	0.2
	2005–2008	104	0.02	28	48.9 (29.9–80.0)	0.27
**amphetamine**	1997–2000	216	0.03	4	2.3 (0.8–6.5)	0.02
	2001–2004	84	0.01	5	9.6 (3.9–23.3)	0.06
	2005–2008	32	0.01	0	0 (0.0–0.0)	0
**no substance**	1997–2000	3988	0.56	32	1	0.01
	2001–2004	9008	0.66	56	1	0.01
	2005–2008	4907	0.7	27	1	0.01

For each substance, the risk of visiting a first aid station was calculated by dividing the number of first aid visits (FAVs) related to that substance by the number of FAVs for that cohort.

CI = 95% confidence interval. Confidence interval for relative risk (RR) of a serious incident (SI) was calculated using Morris and Gardner's [46] formula.

The category *no substance* is the reference category for the SI risk ratios.

**Table 6:** Number and risk of first aid visits and serious incidents associated with using different combinations of substances.

Substances	Period	Nr of FAAs	Risk FAS	Nr of SI	RR SI (CI)	Risk SI
**alcohol+cannabis**	1997–2000	54	0.01	1	2.3 (0.3–16.6)	0.02
	2001–2004	227	0.02	6	4.3 (1.9–9.8)	0.03
	2005–2008	99	0.01	4	7.3 (2.6–20.6)	0.04
**alcohol+GHB**	1997–2000	2	0	0	0 (0.0–0.0)	0
	2001–2004	66	0.01	17	41.4 (25.5–67.3)	0.26
	2005–2008	54	0.01	15	50.5 (28.5–89.4)	0.28
**ecstasy+GHB**	1997–2000	10	0	3	37.4 (13.6–102.4)	0.3
	2001–2004	113	0.01	31	44.1 (29.7–65.7)	0.27
	2005–2008	65	0.01	16	44.7 (25.4–78.9)	0.25
**ecstasy+amphetamine**	1997–2000	340	0.05	5	1.1 (0.7–4.7)	0.02
	2001–2004	63	0.01	1	0.8 (0.4–18.2)	0.02
	2005–2008	22	0	1	2 (1.2–58.2)	0.05
**no substance**	1997–2000	3988	0.56	32	1	0.01
	2001–2004	9008	0.66	56	1	0.01
	2005–2008	4907	0.7	27	1	0.01

For each combination of substances, the risk of visiting a first aid station was calculated by dividing the number of first aid visits (FAVs) rrelated to that combination by the number of FAVs for that cohort.

CI = 95% confidence interval. Confidence interval for relative risk (RR) of a serious incident (SI) was calculated using Morris and Gardner's [46] formula.

The category *no substance use* is the reference category for the SI risk ratios.

## Discussion

Approximately one-third of all rave-party visitors who sought first aid reported having a substance-related problem. Visitors with substance-related problems stayed longer at first aid stations than those without a substance-use problem. Altogether, 515 of 10,100 substance-related incidents were classified as serious, and 16 of these were life-threatening. Most substance-related incidents were associated with ecstasy or alcohol use or both. It is noteworthy, however, that in the Netherlands alcohol use is relatively common, but ecstasy use is not. It is possible that the willingness of rave-party visitors to present themselves at a first aid station with health-related complaints was related to the drug that they used. For example, ecstasy users' [9] need for social contact might have prompted them to seek assistance with minor health-related problems more readily than users of other substances. Additionally, readiness to report one's substance use might have varied according to the social acceptability of using particular illicit drugs.

Unlike what most other recent studies from various countries have found [17]–[24], the occurrence of acute substance-related health problems found in this study was relatively infrequent and the problems were not severe. From their systematic review of the harmful effects of ecstasy use, Rogers et al. concluded that this drug rarely causes death [25], and Chinet et al. reported that party-goers who use drugs appeared to be particularly receptive to harm-reduction measures [26]. It might be concluded, therefore, that harm and risk reduction as practiced in the Netherlands is effective [7],[8]. It should also be noted that the Dutch generally use drugs in moderation, and they avoid using highly risky substances, such as methamphetamine, which are used in many other countries [27]–[34].

In the current research, no evidence was found for life-threatening, acute effects of GHB. Nevertheless, professional medical care is often required after GHB use and the syndrome that can occur (altered consciousness, vomiting, and subnormal body temperature) can be dangerous. Health education should focus on these secondary effects in addition to the primary effects.

Questions remain about whether the relatively low rate of severe incidents that occurred was related to the open nature and legal status of rave parties in the Netherlands. It would, therefore, be important to replicate this study in other countries. To our knowledge, there is no other published research on substance-related incidents that occur during large-scale events. It would be worthwhile for future research to focus on the causes of these incidents [21],[35]–[45]. Finally, we recommend that future research also address the secondary factors related to substance-related incidents and the mechanisms involved in them, such as GHB-related airway threats and hypothermia, ecstasy-related hyponatremia, excited delirium, and the serotonin syndrome.

### Limitations

There were limitations of the current study that should be acknowledged. For example, long-term effects on substance use or drug addiction were not addressed. Although the study sample was large, it included only self-referrals, which might not be representative of all health-related incidents at rave parties. It is possible that many people who experienced negative effects did not present themselves at a first aid station. In fact, Wijngaart et al. and de Bruin et al. reported that some rave party visitors sought help from friends, security personnel, or food-service staff [5],[11],[12]. Substance use at rave parties might be underreported and hence underestimated because stigmatization or a fear of legal involvement. For reasons such as these, some visitors with health complaints may have gone directly to their family physician or a hospital emergency room, rather than visiting an on-the-scene first aid station.

### Conclusions

Only a small proportion of rave-party visitors (0.3%) reported substance-related health problems. The problems that were reported at first aid stations were usually related to ecstasy or alcohol use. Substance users who sought first aid stayed four times as long at a first aid station as nonsubstance users. A total of 515 of the substance-related incidents could be regarded as serious; this amounts to 0.01% of all party visitors, 1.8% of all visitors who sought first aid, and 5.1% of all substance users who sought first aid. Sixteen cases were classified as life threatening. Visitors who used GHB, with or without alcohol or ecstasy, and those who used cocaine were highest on relative risk of having a serious incident. Finally, it should be notes that although lifetime prevalence of GHB use is low, this substance causes many problems.
